# 

**Published:** 1852-01

**Authors:** 


*Transactions of the American Medical Association.* Vol. IV.—This work has been received just as our present number goes to the press. We shall notice it in our next issue.

---

"*Happy New Year.*"—With the commencement of a new year, we respectfully tender to our readers the time-honored salutation of the season. Another year has gone! How short the period since the expiring year was ushered in by the customary greetings! Fortunately for those who honor our pages with a perusal, the printer's devil refuses more copy; else we might yield to the temptation to moralize on the rapid progress of time, the short duration of life, etc.—topics concerning which it is easier to rehearse truisms, than to communicate interesting information. We content ourselves, therefore, with saying to one and all *a happy new year.*

